# Emerging roles and mechanisms of ERK pathway mechanosensing

**DOI:** 10.1007/s00018-023-05007-z

**Published:** 2023-11-10

**Authors:** Flora Crozet, Romain Levayer

**Affiliations:** Department of Developmental and Stem Cell Biology, Institut Pasteur, Université de Paris Cité, CNRS UMR 3738, 25 Rue du Dr. Roux, 75015 Paris, France

**Keywords:** ERK, EGFR, Mechanics, Mechanotransduction, Morphogenesis, Growth, Signalling, Self-organisation, Cancer

## Abstract

The coupling between mechanical forces and modulation of cell signalling pathways is essential for tissue plasticity and their adaptation to changing environments. Whilst the number of physiological and pathological relevant roles of mechanotransduction has been rapidly expanding over the last decade, studies have been mostly focussing on a limited number of mechanosensitive pathways, which include for instance Hippo/YAP/TAZ pathway, Wnt/β-catenin or the stretch-activated channel Piezo. However, the recent development and spreading of new live sensors has provided new insights into the contribution of ERK pathway in mechanosensing in various systems, which emerges now as a fast and modular mechanosensitive pathway. In this review, we will document key in vivo and in vitro examples that have established a clear link between cell deformation, mechanical stress and modulation of ERK signalling, comparing the relevant timescale and mechanical stress. We will then discuss different molecular mechanisms that have been proposed so far, focussing on the epistatic link between mechanics and ERK and discussing the relevant cellular parameters affecting ERK signalling. We will finish by discussing the physiological and the pathological consequences of the link between ERK and mechanics, outlining how this interplay is instrumental for self-organisation and long-range cell–cell coordination.

## Introduction

Cells and tissues are constantly subjected to a wide range of mechanical stresses which they cope with and timely adapt to. This extensive plasticity to environment changing is essential for tissue morphogenesis, homeostasis, and tissue regeneration. This potential is largely achieved through mechanotransduction [1, 2], the ability of cells to probe and convert stimuli from their mechanical environment into biochemical signals that influence cell decision-making, including differentiation, proliferation, and survival. Numerous studies have focussed on characterising mechanisms and pathways mediating stress sensing and intracellular force transduction, providing the cellular and the molecular bases that couple mechanical stimuli to ensuing cellular outcomes. To date, these studies have been restricted to a small number of force-induced pathways, including effectors with a primary sensor role via stress-induced conformational change, or a more downstream involvement in relaying mechanical inputs. These include YAP/TAZ [3–5], stretch-activated Piezo channel [6–8], JNK (c-Jun N-terminal Kinase) [9, 10], β-catenin and Wnt pathway [11, 12] or P53 [13, 14].

Significantly, the ERK (Extracellular Regulated Kinase) pathway is now emerging as an additional player in the mechanotransduction ecosystem. ERK1–2 is a conserved and widely expressed serine/threonine MAPK, and a central integrator of growth factor and mitogen signals that regulate many cellular functions from proliferation, survival, differentiation to cell migration [15]. Its activation is an integral part of a phosphorylation cascade initiated by the phosphorylation of the intracellular domain of membrane RTK receptors upon ligand binding, followed by activation of Ras GTPases, RAF and MEK, leading to the recruitment of a myriad of downstream ERK effectors, with cellular outcomes depending on stimulus/cellular contexts and the dynamics of ERK activity [16]. The connexion between mechanical stress and ERK activity variation was first explored in the late 1990s and early 2000s in a variety of cell cultures. By subjecting cells to different types of mechanical strains and substrate stiffness, and using traditional immunodetection techniques to measure ERK activity, they pioneered the emerging role of ERK as a mechanotransducer [17–25]. Nowadays, the recent and rapid development of new live sensors of ERK activity and imaging techniques has enhanced our current understanding of ERK involvement in mechanotransduction [26]. Notably, development of live single-cell reporters of ERK activity with high sensitivity and versability, initially FRET-based biosensors [27, 28], followed by Kinase Translocation Reporters (KTRs) [29, 30], have enabled qualitative and quantitative single-cell analyses of ERK activity dynamics, with the advantage of subcellular compartment assessment for FRET-based sensors, and higher dynamic range and multiplexed imaging options for KTRs. In parallel, the design of optogenetic tools driving synthetic stimulation of ERK pathway components has led to better understanding of the downstream decoding mechanisms of ERK activity dynamics in multicellular systems, and to the dissection of the signalling step integrating mechanical stress [31–35].

In this review, we discuss the emerging implications of the ERK pathway in mechanotransduction. We first present an overview of in vivo and in vitro contexts that have established a clear link between ERK activity, mechanical stress, tissue/cell deformation and shape and provide a systematic comparison of the mechanical stimuli and timescale associated with ERK variations. We then explore the key cellular parameters sensed, along with the different molecular mechanisms associated with mechanically induced ERK modulation. We then discuss the in vivo physiological and pathological functions of ERK mechanotransduction, highlighting the novel properties emerging from fast modulation of ERK by mechanical inputs. This review can also be complemented by other excellent recent reviews covering the link between ERK mechanics and self-organisation [36–38].

## Characterisation of ERK responses to mechanics in vitro and in vivo

ERK activity has been linked to mechanics in a variety of cellular and tissue contexts and documented to respond to a wide range of mechanical cues. This includes response to cell/tissue strain in different axis and tissue/cell morphology, not only in cell culture, but also in in vivo settings (Table 1). We herein document them as comprehensively as possible, with a concise description of the different perturbative approaches used to trigger mechanical stress. The rationale is to establish whether a general mechanism is shared regarding mechanical modulation of ERK activation.

**Table 1:**
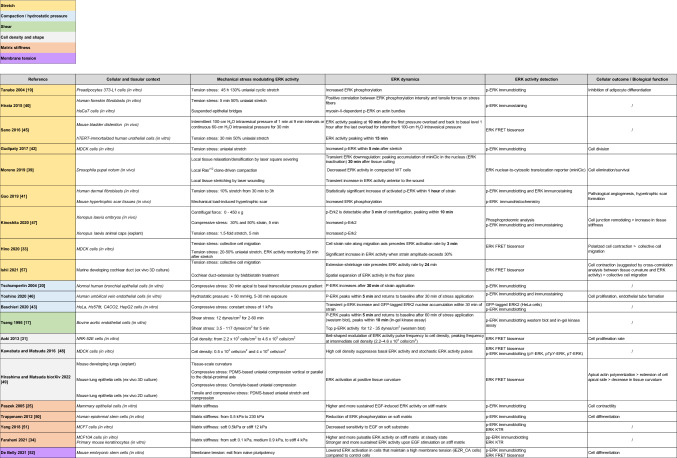
Summary of the studies linking mechanical stress and regulation of ERK activity

Summary of the main relevant parameters outlined in various studies connecting mechanical stress to ERK regulation. This includes the biological context, the type of mechanical perturbations, the characteristic timescales, the methodology to measure ERK variation as well as putative outputs and biological functions

### Mechanical perturbations modulating cell/tissue strain

With regard to mechanically induced cell/tissue deformation, ERK has been reported to respond to the three standard mechanical stresses, namely tension [19, 33, 39–42], compression [20, 39, 43] and shear [17, 18]. Briefly, tensile stress is defined by forces per unit of area generated by outward forces (pulling) that will tend to stretch an object, compressive stress is associated with inward forces (pushing) that will tend to squeeze an object, whilst shear stress is generated by forces applied parallel to the object which will tend to deform a rectangle into a parallelogram.

#### In vitro tissue/cell deformation

**Fig. 1:**
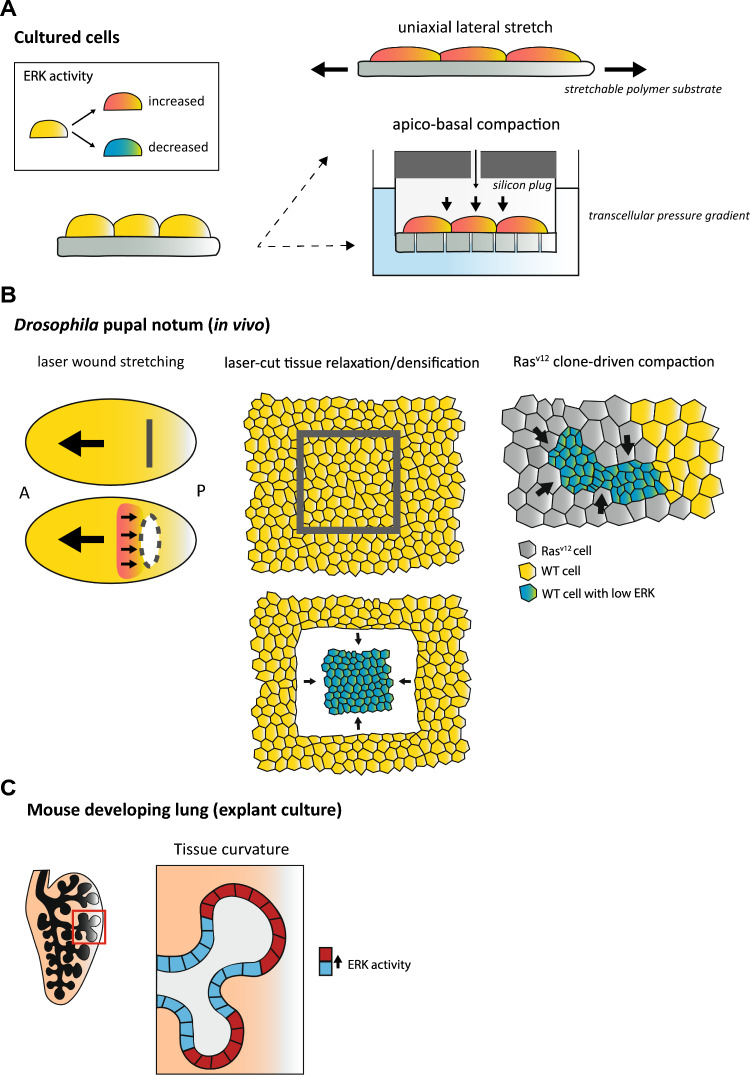
In vitro and in vivo mechanical regulation of ERK activity. **A**–**C** Modulation of ERK activity under mechanical stress is presented in a binary fashion, with cells turning red due to increased ERK activity, and conversely blue due to decreased ERK activity. **A** In vitro application of mechanical strain to cultured cells. Top: cells plated on an extensible polymer substrate are deformed upon lateral uniaxial stretching of the substrate, associated with an increase in ERK activity. Bottom: Bronchial epithelial cells cultured at air–liquid interface on a microporous substrate. Pressure application through a port in a silicon plug places cells in a pressure chamber over the cell apical surface, with the cells experiencing compressive stress via an apico-basal transcellular pressure gradient [20]. Both lateral stretching and apico-basal compaction induce increased ERK activity in epithelial cells. **B** Mechanical perturbations in the *Drosophila* pupal notum [39]. Left: laser wounding of the notum epithelial monolayer triggers local tissue stretching and a transient increase in ERK activity anterior to the wound due to anterior-oriented tissue drift (big arrow) and posterior-oriented wound closure (smaller arrows). A stands for anterior and P for posterior. Middle: Laser cutting of a notum square induces local relaxation/densification of the tissue and a decrease in apical cell surface area, triggering transient ERK downregulation. Right: Compaction of WT cells by increased growth of surrounding Ras^v12^ clones in the context of cell competition progressively reduces ERK activity in compacted WT cells. **C** Tissue curvature of the murine developing lung epithelium instructs ERK activity, which is higher in convex than in concave regions [49]

The connexion established between tension stress and ERK activity modulation has been massively evidenced with in vitro devices featuring stretchable polymer substrates on which cells are previously seeded. Cyclic or constant uniaxial stretch is applied to the elastic substrate to deform the cells, and ERK activity is monitored before and after stress (Fig. 1A top). In these settings, various cell types display ERK sensitivity to tensile stress: from epithelia [33, 42], fibroblasts [40, 41], osteoblasts [44], to preadipocytes [19]. 50% uniaxial stretch of elastic silicon chambers for 30 min triggers a rapid increase in ERK activity (peaking within 15 min) in human urothelial cells [45]. Similarly, a 5-min 50% uniaxial stretch of human foreskin fibroblasts induces ERK activation on stress fibres in a myosin-II inhibition context [40]. In MDCK (Madin–Darby Canine Kidney) cells, an increase in ERK activity is measured after 5 min of substrate stretch (and 1 min for EGFR phosphorylation), and ERK activity gradually rises with the degree of stretch (statistically significant at 30%, 40% and 50% stretch) [33]. Alternatively, a statistically significant increase in phospho-ERK was observed after 1 h of cyclic stretch (10% elongation) in human dermal fibroblasts plated on flexible silicone rubber plates [41]. Although the degree and the duration of deformation vary from one system to another, ERK response to mechanical stretching appears to be a robust cellular property shared by different cell lines in vitro.

Interestingly, ERK activity also responds to negative strain and hydrostatic pressure in cultured epithelial cells. This was evidenced by subjecting human bronchial epithelial cells to apical–basal compressive stress (20 cm H_2_O ~ 2 kPa transcellular pressure), a stress similar to the one experienced during airway constriction in vivo [20]. Compressive stress triggers a ~ 10% reduction in the thickness of bronchial epithelial cell layers and a ~ 87% decrease in the volume of the lateral intercellular space. In this context, 30 min of cell compaction induces strong ERK activation (Fig. 1A bottom). HeLa cells confined under constant compressive stress of 1 kPa (similar to pressure in solid tumours, 0.21–20 kPa) experience a transient increase in ERK activation, statistically significant from 5 to 30 min of stress, with transient nuclear enrichment of GFP-tagged ERK2 [43]. On a similar timescale, hydrostatic pressure of + 50 mmHg (~ 6.7 kPa, emulating the average blood pressure increase during exercise) induces a transient increase in ERK phosphorylation that peaks within 5 min and returns to basal levels after 30 min of stress application in human umbilical vein endothelial cells, which is associated with endothelial tube formation [46]. Thus, pressure of kPa range can trigger ERK activation in various contexts within few minutes.

#### Mechanical stresses experienced in vivo

Whilst some in vitro devices mimic the mechanical stresses that cells would experience in a physiological/pathological context, modulation of ERK activity by tissue/cell strain perturbations has also been evidenced in various in vivo contexts. In the developing *Drosophila* pupal notum (a single layer epithelium in the thorax), local stretching/compaction of the epithelial monolayer transiently increases/downregulates ERK activity (assessed by the nucleus accumulation of the ERK sensor miniCic), which subsequently modulates cell survival (Fig. 1B) [39]. This was on the one hand demonstrated by ectopic stretching driven by tissue stitching to the cuticle through laser wounding, which triggers cell stretching and ERK activation anterior to the wound due to global tissue drift towards the anterior side and cell immobilisation on the posterior side (Fig. 1B left). Conversely, releasing pre-stress by a square pulsed-laser cut of the tissue induces rapid relaxation/densification of the tissue and a transient downregulation of ERK activity (with a minima reached ~ 30 min after cutting) (Fig. 1B middle). Finally, local tissue compaction driven by the overgrowth of Ras^V12^ clones progressively downregulates ERK activity in WT-compacted cells, leading to WT cell elimination and clonal expansion (Fig. 1B right). In the context of notum development, local positive and negative tissue strains are therefore associated with opposite ERK activity outcomes that instruct whether cells survive or die. This places the ERK pathway in a central position in the mechanical fine-tuning of cell elimination.

In vertebrates, bladder distention by elevated intra-vesical pressure activates ERK in mouse urothelial cells [45]. These observations were permitted by intravital imaging of the mouse bladder, and intra-vesical pressure applications, with a FRET sensor to monitor ERK activity. In this respect, application of intermittent 100-cm H_2_O intra-vesical pressure (~ 10 kPa) for 1 min at 9-min intervals is sufficient to rapidly activate ERK in urothelial cells (peaking at 10 min after the first pressure overload and returning to basal levels 1 h after pressure release). Moreover, continuous 60-cm H_2_O (6 kPa) intra-vesical pressure application for 30 min, mimicking bladder urinary retention, induces strong ERK activation in the urothelium [45], illustrating the physiological relevance of this pressure-induced ERK activation.

In *Xenopus laevis* embryos, ectodermal cells activate ERK2 during gastrulation, during which the cells are allegedly exposed to tensile forces induced by gastrulation movements [47]. Centrifugation of *Xenopus* embryos triggers embryo deformation, from spherical to flattened, which enhances ERK activity. Phospho-ERK2 is detectable as early as 3 min after centrifugation and peaks within 10 min. Similarly, subjecting embryos to compressive stress or stretching animal caps explants for 5 min leads to embryo deformation that promotes ERK2 phosphorylation, illustrating that ERK is an early target of mechanical force during *Xenopus* embryogenesis.

### ERK regulation by tissue/cell shape and density

We have reported so far ERK modulations triggered by mechanical perturbations of tissue/cell strain. ERK activity has also been documented to respond directly to steady-state tissue/cell shape and density.

#### Cellular density

A clear link has been established between modulation of ERK activity dynamics and cell density in epithelial cells, correlating with the rate of cell proliferation and the sensitivity of ERK activity to upstream oncogene proteins. Cultured NRK-52E (Normal Rat Kidney 52E) and MDCK cells exhibit heterogeneous stochastic ERK activity pulses whose frequency varies as a function of cell density in a bell shape [31, 48]. ERK peak is observed at an intermediate density of 2.2–4.6 × 10^4^ cells/cm^2^ for NRK-52E cells (which vary between 2.2 × 10^3^ cells/cm^2^ and 4.6 × 10^5^ cells/cm^2^ at confluence) [31]. Interestingly, a similar density in MDCK cells (4 × 10^4^ cells/cm^2^) abrogates stochastic ERK activity pulses [48], suggesting that the quantitative relationship between density and ERK is cell type-dependent. Importantly, the induction of active Ras and BRaf in MDCK cells does not bypass ERK activity suppression by high cell density, suggesting that density sensing acts relatively downstream in the pathway. This is another illustration of the context-dependant outcome of oncogene activation. Thus, cell density can directly modulate the frequency of ERK pulses which will eventually modulate the rate of cell proliferation [31].

#### Tissue curvature

Recently, Hirashima and Mastuda demonstrate that ERK activity also correlates with tissue-scale curvature [49]. In the lobes of developing murine lungs, epithelial ERK activity displays a distinct spatial distribution, with higher activity in convex regions where the basal cell area is larger, and low activity in concave regions where basal area is small, suggesting an interplay between ERK activity and cell morphology (Fig. 1C). This spatial pattern is also visible in 3D culture of isolated lung epithelial tissue embedded in FGF1-containing Matrigel, suggesting that it is not driven by enhanced ligand secretion in convex regions. Live imaging ex vivo outlined a spatio-temporal correlation between decrease of tissue curvature at the tip apex and decrease of ERK activity with an averaged delay of 97 min. Furthermore, local modulation of tissue curvature through parallel or vertical compression using PDMS or hydrostatic pressure was also sufficient to modulate ERK activity.

### Subcellular force modulation regulating ERK activity

We have so far restricted our attention to the modulation of ERK activity upon global modulation of cell/tissue strain and shape. This section focuses more specifically on mechanical stress at the subcellular level that regulates ERK activity. In this regard, ERK activity has been associated with stress sensing at the cell–substrate interface via changes in underlying matrix stiffness [25, 34, 50, 51], forces conveyed by cell–cell junctions in the context of collective cell migration [33], to modulation of cell membrane tension associated with cell differentiation [52].

#### Cell–substrate interface

ERK activity has been shown to respond to mechanical stress applied at the cell–substrate contact and specifically associated with subcellular compartments at the interface with the substrate. The most obvious link between cell–substrate interaction and ERK was first illustrated through the impact of substrate stiffness. The finding that the dynamics of ERK activity can be mediated by matrix stiffness is particularly relevant in the context of tumorigenesis. Matrix rigidity is a common feature of the solid tumour microenvironment during tumorigenesis and induces modulation of various tumour cell outcomes, such as tissue invasion and proliferation [53]. The link between substrate rigidity and variation in ERK activity has been established by culturing mammalian cancer cell lines on matrices with different elastic moduli, ranging from healthy tissue stiffness (0.1 kPa) to stiffness of rigid mammary tumours (around 4 kPa). Several studies demonstrated that stiff matrices increase the frequency of EGF-induced ERK activity pulses and foster a shift towards more sustained and higher ERK activity [25, 34, 51]. In MCF7 cells (Michigan Cancer Foundation 7), cells cultured on a soft 0.5 kPa or rigid 12 kPa substrate display a different response sensitivity to EGF stimulation, which is decreased on soft substrates. In addition, fewer pulses of ERK activity are measured on soft versus rigid substrates for the same EGF concentration stimulation, as indicated by the frequency of ERK KTR nuclear exit [51]. In MCF10A cells cultured on fibronectin-coated polyacrylamide hydrogels, the stiff matrices foster a higher and more pulsatile ERK activity at the steady state, and a stronger ERK activity shifted towards sustained signalling upon EGF stimulation [34]. Similarly, MEC cells exhibit a higher and more sustained EGF-induced ERK activity on stiffer matrices, associated with enhanced cell contractility [25]. Substrate rigidity also plays an instructive role in stem cell fate through modulation of ERK activity, as shown by the differentiation of human epidermal stem cells associated with decreased ERK activity on soft polyacrylamide substrates [50]. Altogether, these works suggest that soft substrate acts as an attenuator of ERK activity, which influences downstream cell contractility and fate.

ERK activity is also coupled to subcellular structures located on the basal part of the cell, which may act as sensors of substrate stiffness and strain, and modulate the dynamics of ERK activity. In human foreskin fibroblasts, phospho-ERK colocalises with stress fibres in a myosin-II and tension-dependent manner [40]. Uniaxial substrate stretching after myosin-II inhibition treatment increases P-ERK recruitment specifically on more tensed stress fibres parallel to the stretching axis. Individual stress fibres thus act as mechanosensors for ERK activation, which may be relevant for locally directing ERK signalling to precise subcellular sites. ERK activity is also coupled to dynamic cellular protrusions which is further enhanced on stiff matrix stiffness [51]. In MDCK cells, the specification of leader cells during collective migration is mediated by persistent ERK activation, promoted by lamellipodia at the free edges and a positive feedback loop between HGF receptor activation and cell protrusion [54].

#### Cell–cell interface

In addition to ECM–cell interaction, ERK is also modulated by mechanical forces transmitted at the cell–cell interface through adherens junctions. In collective cell migration, a leading edge of cells transmits guided mechanical forces to following cells in an orderly, coordinated fashion, promoting directional cell migration and tissue expansion associated with morphogenesis and tissue repair [55]. In this context, waves of ERK activity were observed in the opposite direction to cell migration in MDCK monolayers, mouse cochlea and mouse epidermis [33, 56, 57]. In MDCK cells, ERK activation triggers polarised rear contraction, which induces the pulling and the stretching of the neighbouring following cells, propagating ERK activity and the migration unidirectionality on a large scale in a self-sustaining manner [33]. Mechanical coupling through adherens junction is essential for ERK wave propagation and long-range coordination of cell migration [33]. The parameters and the properties of this coordination will be discussed in greater detail in the third section of the review. During MDCK cell collective cell migration, cross-correlation analysis reveals that cell strain rate along the migration axis (evaluated by particle image velocimetry) precedes ERK activation rate by 3 min, outlining a rapid impact of cell deformation on ERK signalling [33]. Retrograde ERK waves were also described in 3D ex vivo culture of the developing murine cochlear duct opposite to the base-to-apex global tissue flow [57]. Here, the extension–shrinkage rate precedes ERK activity rate by 24 min [57]. Whilst these numbers are mostly based on cross-correlation and should be interpreted with caution given the complex temporal pattern generated by the mutual regulation between ERK and cell mechanics, these differences are nonetheless interesting and may be due to different characteristic cell size (4 µm for cochlear duct cells compared to 20 µm for MDCK cells, in line with the slower ERK wave propagation speed in the cochlear duct), difference between 2 and 3D culture, or differences in the upstream signal (FGFR-dependent in the developing cochlea channel, and EGFR-dependent for MDCK cells).

#### Membrane tension modulation

Global modulation of membrane tension has also been associated with ERK regulation. The spreading of mouse embryonic stem cells on an extracellular substrate is concomitant with a decrease in plasma membrane tension and an increase in ERK endosomal activity, hence promoting cell fate transition and exit from naïve pluripotency [52]. Tension reduction is prompted by a decrease in ERM (Ezrin Radixin Moesin) protein phosphorylation driven by a β-catenin-mediated reduction of RhoA activity*.* During the exit from pluripotency, cells that maintain high membrane tension by expressing a constitutively active form of Ezrin have a reduced global ERK activity compared to control cells, reduced ERK activity at early endosomes, as well as deregulation of ERK-targeted genes evidenced by RNA-Seq.

In conclusion, co-option of ERK signalling appears to be a general mechanism deployed by cells subjected to mechanical stress. ERK activity is regulated by the three standard mechanical stresses (tension, pressure and shear), whether extrinsic or intrinsic in origin, together with tissue/cell morphology. In some cases, ERK activity gradually rises as the degree of mechanical stress increases [33, 47], whilst in others, it adapts in a biphasic manner, with a maximised frequency of ERK activity pulses at intermediate stress [31]. In most of the cases, the most relevant mechanical/geometrical input remains hard to identify (e.g.: strain rate versus absolute shape, cortex versus membrane tension, apical area versus cell height, see [58] for a similar discussion). However, some commonalities can be found in apparently opposite mechanical perturbations. For instance, centrifugation, compaction and stretching result in similar ERK activation outputs in *Xenopus* embryos most likely because they all lead to cell flattening and spreading [47]. Similarly, epithelia lateral stretching [33] and apico-basal compaction [20] both lead to ERK activation and an increase of cell apical area relative to cell height, and conversely for epithelial cell lateral compaction [39] (Fig. 1A). This is also in good agreement with the strong reduction of ERK pulse frequencies at high cell density, associated with low apical area and high cell height [31]. This may point to a universal role of cell shape (high apical/basal area relative to cell height) with squamous-like shape being more permissive for ERK activation relative to columnar shape. Furthermore, although the cellular systems, the type of mechanical stress and cellular outcomes differs, most of these studies outline similar short timescales of ERK modulation, in the order of few minutes (Table 1). This suggests the existence of regulatory mechanism based on protein–protein interaction/protein conformation/phosphorylation, rather than gene expression-based regulation. This prompts us to review the cellular and the molecular mechanisms that convey mechanical stress to the modulation of ERK activity.

## Cellular and molecular mechanisms of ERK mechanical regulation

**Fig. 2:**
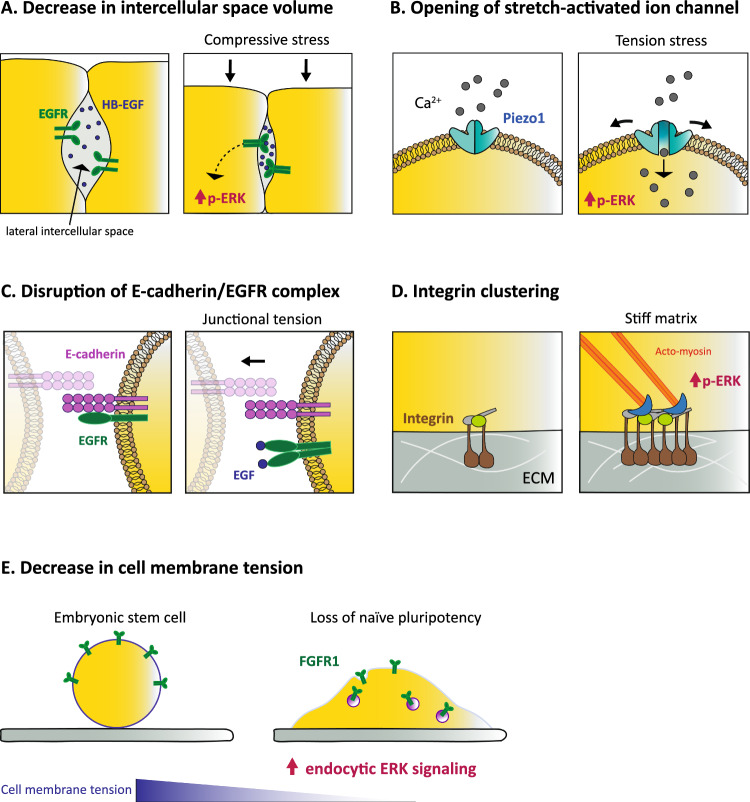
Cellular and molecular mechanisms coupling mechanics to ERK activity. **A** Apico-basal compaction of epithelial cells reduces the volume of the lateral intercellular space, increasing ligand concentration and triggering EGFR-ERK signalling [20]. **B** Mechanical stretch induces the opening of the stretch-activated Piezo1 channel and an influx of calcium activating ERK [42]. **C** EGFR monomer and E-cadherin homodimers form a heterotrimeric complex at the plasma membrane that sequesters EGFR monomer. Forces applied to homophilic E-cadherin bonds disrupts the complex, enabling EGFR dimerization and signalling upon ligand binding [65]. **D** Matrix stiffness induces integrin clustering and focal adhesion assembly, enhancing ERK activation [25]. **E** Decreased cell membrane tension during early differentiation of embryonic stem cells increases FGFR1 endocytosis and endocytic ERK signalling [52]

Having described the different contexts associating cell/tissue deformation and shape with ERK activity, we now explore the molecular mechanisms by which mechanics drives variation in ERK activity. In particular, we review the key parameters of cellular sensing, and their associated putative molecular mechanisms inducing ERK signalling. So far, no consensus has been reached on a single regulatory mechanism, suggesting the existence of multiple mechanisms, each of which has been substantiated in their associated experimental system (Fig. 2). Two common mechanisms have been documented to date: (1) an indirect contribution of mechanical stress to ERK variation via sensing of cell/tissue shape and extracellular space geometry; (2) a direct sensing of cell mechanics modulation. We conclude this section by discussing the epistatic link between mechanics and ERK modulation and identifying the core components of the pathways that can be modulated by geometry/mechanical inputs.

### Sensing of tissue/cell shape and intercellular space geometry

It has been well documented that cell geometry and surface of the cell–cell interface can modulate the output of signalling pathways [59–61]. For instance, cell density and associated cell shape modulation polarise the localisation of TGF-β receptors on the cell basal side, altering TGF-β signal transduction [62]. The same applies to changes in the volume of the intercellular space, which can increase the concentration of available soluble factors and binding to membrane receptors. Here we describe two mechanisms by which mechanical stress modulates cell/tissue geometry or the shape of the intercellular space, fostering enhanced ERK activity.

#### Change in intercellular space volume

In bronchial epithelial cells, ERK activation depends on the shedding of EGF family ligands from the basolateral surface, as inhibition of matrix metalloprotease activity reduces both baseline and compaction-induced ERK phosphorylation [20]. Alongside, the application of apico-basal compressive stress results in a ~ 10% reduction in cell layer thickness, which is not due to a significant reduction in total cell volume, but to a sharp decrease (87%) in the volume of the compliant lateral intercellular space (Fig. 2A) [20]. The study predicts that with a constant ligand shedding rate, the concentration of EGF family ligands in the intercellular volume is inversely proportional to the width of the lateral intercellular space, thus leading to an approximately eightfold increase in effective ligand concentration following a 10% reduction of cell thickness. This matches the increase in HB-EGF ligand required experimentally to mimic strain-induced ERK phosphorylation. Thus, tissue geometry/compression can be encoded in variation of the lateral intercellular space which will be sensed through the modulation of EGFR ligand concentration.

#### Cell morphology sensing

Variations in cell geometry, such as changes in cell aspect ratio and apical and/or basal area, could also alter the number of transmembrane receptors that can bind extracellular ligands, thus modulating downstream signal transduction. This would be relevant in conditions where ligands or receptors are specifically enriched on one specific side of the cell (apical, basal or lateral). In the lobes of developing murine lungs, whilst all FGFR are expressed homogenously, FGF1 is produced by mesenchymal cells surrounding the epithelial basal side [49], suggesting that FGFR activation may be restricted to the basal side. Accordingly, convex regions with larger cell basal area also display high ERK activity. Moreover, FGF1 is endocytosed specifically at the basal surface of positive curved regions during ex vivo culture in Matrigel with homogeneous FGF1. This is all in agreement with a curvature sensing mechanism based on modulation of cell basal surface and FGFR ligand accessibility. However, nothing excludes at this stage a contribution of curvature-dependent difference in membrane tension which could also modulate ERK activation.

#### Direct sensing of changes in cell mechanics

In addition to the indirect perception of mechanical cues by cell and tissue shape, direct sensing of cell mechanics can be transmitted intracellularly to signalling pathways via force-induced conformational/state changes in mechanosensory proteins [1]. In this respect, force changes at cell adhesion sites or changes in cell membrane tension translate then biochemically into set points for ERK signalling. This was reported for force-sensitive ion channels [42], integrins and focal adhesion components [25, 41], as well as actin bundles operating as signalling platforms [40].

#### Stress sensing through increased transmembrane channel flow

In MDCK cells, rapid activation of ERK upon stretching depends on the stretch-activated Piezo1 channel and calcium influx (Fig. 2B) [42]. MDCK cells stretched at steady state by wounding or with a uniaxial stretching device result in a rapid increase in Piezo1-mediated cell division, as validated by Piezo1 knockdown and GFP-tagged Piezo1 rescue, as well as pharmacological calcium inhibition. The stretch-induced increase in ERK phosphorylation in this context is abrogated when cells are incubated with gadolinium, a non-specific inhibitor of stretch-activated channels. This places Piezo1 signalling in a pivotal position in the stretch-sensing mechanism, instructing a rapid increase in ERK activation, which enhances cell division of early G_2_ cells. Similarly in endothelial cells, transient ERK activation under hydrostatic pressure also relies on force-induced transmembrane channel flow [46]. Transient ERK activation by hydrostatic pressure is mediated by water efflux via the aquaporin 1 channel, which induces a decrease in relative cell area and activation of protein kinase C/Ras-ERK pathway via specific G protein-coupled receptors (α1-adrenergic receptor and serotonin receptor type 2A) [46]. Interestingly, lateral cell stretching and hydrostatic pressure are both sensed by transmembrane molecular/ionic flux, triggering a rapid (5 min) increase in ERK activity [42, 46]. This sensing mechanism appears relevant to the rapid co-option of ERK signalling, which could be suitable for short time-scale stress.

#### Sensing of cell membrane tension via modulated receptor endocytosis

In addition to stretch-activated ion channels, cell membrane tension can be sensed by a modulation of endocytosis of growth factor components, altering downstream ERK signalling. A close interplay has indeed been reported between membrane tension and clathrin-mediated endocytosis [63]. In mouse embryonic stem cells, the exit from naïve pluripotency is mediated by FGF-ERK signalling via a drop in effective membrane tension (Fig. 2E) [52]. Decreased membrane tension in this setting induces increased levels of endocytosis, assessed by a fluid phase uptake assay with pH-sensitive fluorescent dextran, whilst high membrane tension antagonises endocytosis. FGFR1 internalisation is reduced in cells of high membrane tension expressing a constitutively active form of Ezrin, whilst Rab5a overexpression raises receptor internalisation to levels similar to those in WT cells. Consistently, pharmacological reduction of endocytosis rate decreases ERK activity in early endosomes (assessed by a FRET sensor of endosomal ERK activity) and Rab5a overexpression-mediated increase in endocytosis enhances ERK activity independently of membrane tension. In embryonic stem cells, cell membrane tension thus acts as a regulator of cell fate transition by directing FGF receptor endocytosis and endosomal ERK signalling, therefore instructing whether cells remain in a naive state or undergo early differentiation [52].

Conversely in HeLa cells, mechanical stress is also sensed via frustration of clathrin-coated endocytosis, where unresolved endocytic buddings form Clathrin-Coated Structures (CCS)—also named Clathrin plaques— can operate as signalling platforms [43]. Cell compaction drives a global increase in the lifetime of CCS and rapid recruitment of EGFR and HGFR to CCS, followed by transient ERK activation [43]. Similar CCS dynamics and EGFR/HGFR trapping was observed on stiff substrates in HeLa cells on the membrane side facing the substrate [64]. CCS would thus prevent the limited number of activated receptors from being internalised, and potentiate their signalling, ensuing reliable ERK signalling under stress. Although cell membrane tension was not directly assessed in these studies, the compression-induced increase in cell area and plasma membrane blebs are consistent with an increase in membrane tension upon compaction [43]. Thus, membrane tension and receptor endocytosis can have opposite effect on ERK signalling. In one case, the decrease in membrane tension and the subsequent increased receptor endocytosis enable ERK signalling localised to endosomes [52], whilst in the other situation, increased membrane tension and impaired endocytosis would potentiate receptor signalling on local membrane platforms [43, 64]. Differences in the EGFR/HGFR trafficking routes (e.g.: recycling versus degradation) and resident time in the various endosomes could explain these opposite effects. Investigating how the mechanical environment modulates the trafficking route of receptors could be a promising line of future research.

#### Stress sensing involving integrins and focal adhesion components

Stress conveyed by the extracellular substrate is sensed by force modulation at the ECM/cell interface, involving integrins and focal adhesion components. In dermal fibroblasts, increase in ERK phosphorylation upon tension stress is mediated by focal adhesion kinases (FAK) [41]. FAK phosphorylation increases upon 10% stretch, and FAK inhibition decreases stretch-induced ERK activation. In mammary epithelial cells, matrix rigidity is sensed via integrin clustering and focal adhesion assembly/stabilisation (Fig. 2D) [25]. A stiff substrate promotes integrin clustering, FAK phosphorylation (FAKpY397), vinculin recruitment to β1 integrin adhesions, and EGF-induced ERK activity. These results are substantiated using β1-integrin mutants with enhanced transmembrane associations, resulting in larger integrin adhesions and increased EGF-induced ERK activation on soft substrates. Furthermore, in MCF7 cells, the stiff substrate is sensed by localised protrusions which operate as mechanical and growth factor integrators that regulate ERK dynamics [51]. Protrusion generation is an integral part of a positive feedback loop involving coordinated Ras-PIK3 signalling, actin polymerisation, and cellular adhesion that drives ERK activity pulses. Importantly, inhibition of protrusion formation by latrunculin and inhibition of FAK both attenuate matrix stiffness-dependent ERK activity [51]. Protrusions and focal adhesion components therefore fulfil a key function in probing the mechanical environment and converting it into dynamics of ERK activity.

### Epistasis of ERK pathway modulation by mechanics

We have so far reviewed mechanical stress/inputs as well as putative molecular mechanisms of force and shape sensing that will eventually affect ERK. We will review now which components of the pathway upstream of ERK are affected by mechanics and discuss the epistatic relationship between mechanical inputs and ERK pathway.

#### Growth factor ligand–receptor interaction

Although various cellular parameters can be sensed to modulate ERK activity upon mechanical stress, several studies converge to a central role of receptor–ligand interaction modulation. This regulation could be achieved by a variety of receptor-based mechanisms, including variations in EGFR expression levels [34], ligand shedding [33], ligand–receptor binding and internalisation [34, 52] or receptor subcellular localisation/trapping [43, 64, 65]. An example of this regulation at the very apex of the ERK pathway is the modulation of ERK activity dynamics as a function of matrix stiffness. In MCF10A cells, changes in the mechanical microenvironment are sensed at the extracellular, but not intracellular, section of the EGFR-ERK pathway [34]. This was elegantly demonstrated using synthetic optogenetic stimulation of different nodes of EGFR-ERK signalling and testing their sensitivity to substrate stiffness. Synthetic EGFR activation by blue light-induced clustering and autophosphorylation of EGFR cytosolic domains, as well as downstream light-induced activation of Ras, lead to similar sustained ERK activity irrespective of substrate stiffness (which normally modulates both the strength and sustainability of ERK activity, see "Introduction"). Accordingly, the attenuation of ERK signalling by a soft matrix relies largely on the reduction of EGF membrane binding and EGFR activation and, to a lesser extent, on the modulation of EGFR expression levels [34]. Similarly, ERK activation upon stretching is also abolished upon EGFR depletion in the *Drosophila* pupal notum [39] and in MDCK cells [33], suggesting that the modulation of the pathway operates upstream or at the level of the membrane receptor.

Sequestration/release of growth factor receptors from complexes localised to the plasma membrane is an additional mechanism that links mechanical stress to signalling. As mentioned above, recruitment of EGFR to clathrin-coated structures upon cell compaction provides a platform for potentiating signalling [43, 64]. Conversely, the release of EGFR trapped in heteroreceptor complexes, such as E-cadherin-EGFR complexes, drives EGFR signalling upon mechanical stress [65]. Several studies have indeed addressed the epistatic link between E-cadherin and EGFR related to mechanics [65–68]. In multilayered epithelia, E-cadherin spatiotemporally regulates EGFR activity and localisation at the tissue level in a tension-dependent manner [67]. Additionally, it has been documented that monomeric EGFR can be trapped in a heterotrimeric complex of E-cadherin homodimers at the plasma membrane (Fig. 2C) [65]. In this framework, forces applied to homophilic E-cadherin bonds disrupt the complex, releasing EGFR monomers and enabling their dimerisation and signalling upon EGF binding (Fig. 2C). E-cadherin could be considered an integral part of ERK mechanotransduction, directly transducing forces to the E-cadherin–EGFR complex upon mechanical stress and bridging junction tension to EGFR-ERK signalling.

Interestingly, ERK signalling under mechanical stress could also be triggered in a ligand-independent manner. In the ectoderm of *Xenopus laevis* embryos, mechanical ERK2 activation is mediated by FGFR in a FGF-independent manner [47]. In fibroblasts, although not showed to be linked to ERK, the EGFR activity required for rigidity sensing on stiff substrates could be enabled in the absence of ligand by Src-dependent activation [69]. Concordantly, in HeLa cells, Piezo1 activation results in non-canonical serine phosphorylation of EGFR in an SFK-p38-dependent manner, associated with cellular outcomes different from the canonical ligand-dependent signalling [70]. This provides an engaging paradoxical framework in which mechanical forces can both promote receptor–ligand interaction and unconventionally bypass the ligand requirement for receptor activation.

#### Towards more downstream activation of ERK signalling?

Other results point in the direction of a more direct and downstream regulation of ERK activity. In fibroblasts, quantification of tension and P-ERK on individual stress fibres reveals a quasi-linear tailoring of ERK phosphorylation to tension [40]. Although the mechanism of ERK recruitment and activation on stress fibres is not described, it is possible that the requirement for ligand–receptor interaction is bypassed in this context, which could be consistent with a putative direct modulation of ERK by mechanics.

Collectively, the above studies point to a plethora of cellular and molecular mechanisms that regulate ERK activity under mechanical stress. The underlying conundrum is to determine which key cellular parameter is sensed during stress and translated molecularly into ERK signalling. This is a complex issue to resolve, as variables, such as cell shape and mechanics, are closely linked and difficult to uncouple experimentally. By altering cell shape, cell mechanics can be affected accordingly, making the aetiology of ERK signalling difficult to elucidate. The variety of mechanism of ERK regulations observed in different cellular and stress setting may illustrate different options that have been co-opted as the most adaptive for a given perturbation. This underlines the versatile and the plural nature of ERK signalling whose downstream decoding also leads to a wide range of cellular responses and biological functions.

## Physiological and pathological functions of ERK mechanosensing

We have discussed so far the various conditions during which ERK activity can be modulated by cell shape and mechanical stress. What are then the novel tissue/cell properties than can be generated by the connexion between ERK and mechanics? ERK pathways have numerous targets, which through phosphorylation or transcriptional regulation can affect key cellular properties, from the cytoskeleton and cell morphology [71], cell differentiation [72, 73], cell cycle regulation/cell division [31, 73–75], to cell survival [76–79]. As a result, the whole repertoire of ERK targets may be influenced by its connexion with cell shape and cell mechanics. Rather than listing the physiological functions of ERK mechanosensing, we will discuss here the new properties that are directly based on ERK mechanosensing trying to outline the one that are specific to ERK mechanosensing dynamics (relative to other mechanosensitive pathways). This will include the role of ERK mechano-chemical feedback for long-range cell–cell coordination and its role in self-organisation and the spontaneous emergence of spatio-temporal pattern. Eventually, we will discuss how the same modules can be co-opted to promote the emergence and evolution of pathologies.

### Mechano-chemical feedback and long-range coordination

**Fig. 3:**
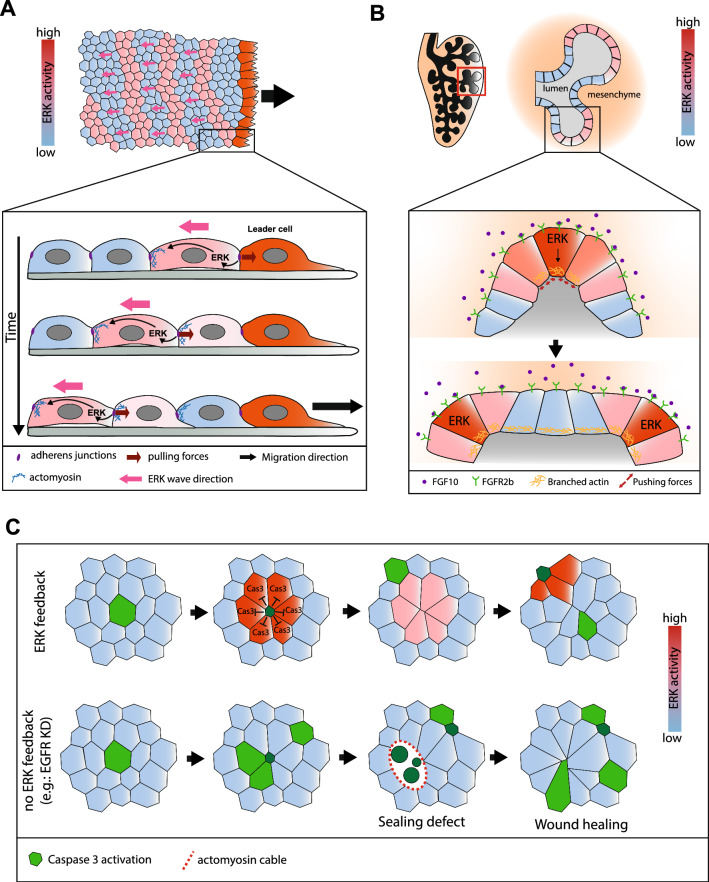
Physiological roles of ERK mechanochemical feedback. **A** ERK mechanochemical waves during collective migration. Schematic of a MDCK layer undergoing collective migration towards the right side (black arrow). Blue to red represents ERK activation levels. High-persistent ERK activity is present at the leader front, whilst ERK waves (pink) propagate towards the back size (opposite direction to migration). Bottom scheme shows the detailed mechanism of ERK wave propagation. Cell stretching promotes ERK activation, which activates cell contractility and actomyosin recruitment towards the rear side (blue) whilst reducing the coupling with the substrate. Propagation of mechanical forces through adherens junction (purple) stretch the neighbouring cell which will then activate ERK. ERK wave propagation also leads to long term polarisation of cells towards the migratory front. **B** ERK curvature sensing and branching morphogenesis in the lung. Schematic of the embryonic mice lung. The alveola (right) is composed of the epithelial layer covering the lumen (grey) and the mesenchyme on the basal side (pink). ERK activity levels (blue to red) correlates with tissue curvature (more activity in convex regions and cell having a larger basal surface). Bottom scheme provides mechanistic details: FGF10 (purple dots) is homogenously produced by mesenchymal cell and preferentially activates ERK in cells with a high basal surface. ERK activation promotes branched actin polymerisation on the apical side which extends apical area (whilst reducing basal surface due to volume conservation). This flattens locally the tissue and increases curvature in the two neighbouring regions which will then activate ERK and initiate two new buds. **C** The role of ERK in cell death distribution regulation in the *Drosophila* pupal notum. Epithelial cell extrusion (green cells, caspase activation) stretches neighbouring cells, this correlates with ERK activation and a transient inhibition of Caspases, hence inhibiting transiently apoptosis and extrusion in the neighbouring cells and preventing the elimination of cells in clusters. The feedback is responsible for a more disperse spatio-temporal distribution of cell death. Without ERK pulses (bottom, EGFR depletion), cell death distribution becomes random. Clusters of cell death can appear by chance which prevents proper extrusion and generates transient holes in the tissue (sealing defect) which are resorbed by wound healing

Complex spatio-temporal dynamics of ERK were described in various cell lines and in vivo. This includes cyclic waves propagating over large spatial scales. ERK waves were first described during collective migration of MDCK cells with cyclic waves moving away from the migrating front up to one mm with a period of 50–90 min and moving at a speed of roughly 2 µm/min [33, 54, 56, 80, 81]. These long-range propagations are driven by the coupling between cell stretching, which activates ERK after few minutes, which then activates cell contractility and MyoII through ROCK after 12 min, thus stretching the neighbouring cells and propagating ERK activation [33, 82] (Fig. 3A). These long-range propagating waves are essential to coordinate collective migration by aligning cell polarity and protrusions opposite to wave’s propagation direction over large distances. Accordingly, drug inhibition of EGFR or MEK, as well as inhibition of ADAM-17 (a metalloprotease required for pro-EGFR ligand release from the membrane) inhibit wave formation and significantly reduce migration speed [33, 56]. Recent work outlined the redundant role of several EGFR ligands for ERK waves, including a predominant role of EGF, but also a contribution of HBEGF, TGFα and EREG (Epiregulin) [83]. Importantly, the collective migration and the global alignment of cell polarity could be rescued despite inhibition of MMPs or EGFR through the generation of artificial ERK waves by optogenetics, with an optimum for waves travelling at a similar speed to the physiological waves (~ 2 µm/min), whilst global ERK activation did not elicit collective movements. Similar ERK waves were also observed in vivo during wound healing in the mouse epidermis with similar propagation speed [84], or during the elongation of the cochlea, where propagation is slightly slower (0.42 µm/min) [57], and are both associated with long-range collective migration opposite to wave propagation. These results suggest that long-range propagating ERK waves are essential for large-scale coordination of cell migration in various contexts. Which properties of the system are required for this long-range coordination and global tissue polarisation? A recent combination of theory and experiments explored more systematically the properties of mechanochemical ERK waves. Using a 1D model encapsulating tension, traction forces, polarity and the mutual feedback between ERK and junctional tension was sufficient to recapitulate quantitatively ERK wave properties (length scale of 7–16 cells, period of 50–90 min, speed of 2 µm/min). This was performed using only three characteristic times estimated experimentally: the delay between cell deformation and ERK activation (4–8 min), the delay between ERK activation and neighbouring cell stretching (100–140 min), and the characteristic time of mechanical stress dissipation through friction (5–16 min, which defines the length scale of deformation propagation). How to explain then that cells behave differently to the front and the back of ERK waves, in other words, how does the system break symmetry and drive unidirectional cell polarisation? The authors first included a polarity term aligning cell polarisation along tension. Symmetry breaking emerges then from the phase difference between ERK activity and mechanical stress, combined with negative impact of ERK on the coupling between stress and polarity. As such, cells are relatively insensitive to the incoming mechanical wave whilst responding strongly to the decreasing phase of the wave, thus breaking symmetry and promoting movement opposite to wave propagation. Of note, this allows global polarisation without long-range gradient and pre-existing polarising cues. Importantly, the model predicts that optimal polarisation will occur for a range of wave properties perfectly matching experimental measurements (predicted length scale of 10–35 cells, period of 2 to 7 h, versus waves of 20 cells and 1–5 h measured in MDCK cells [33, 56, 84]) and the optimal wave speed characterised with optogenetics (2 µm/min [56]). Altogether, this suggests that the parameters of ERK mechanochemical waves are optimised for long-range polarisation and coordination. Importantly, since these wave parameters are set by the characteristic time delay between mechanical stress and ERK modulation (few minutes), this may nicely explain why ERK pathway (rather than other mechanosensitive pathways) may have been co-opted for this long-range coordination.

ERK waves were also described in other physiological contexts, whilst not formally connected to ERK mechanosensation. For instance, ERK waves were described in vicinity of MCF10A oncogenic cell expressing active BRAF, MEK or apoptotic cells [85]. These waves are also mediated by release of EGFR ligands (most likely AREG) through ADAM17 metalloprotease and promote convergent migration of WT cells towards the oncogenic/dying cells, hence facilitating aberrant cell extrusion. However, these waves propagate at a much more limited range (few cells raw) compared to the mechanochemical waves. Similarly, ERK waves were also described during tracheal cell invagination in the *Drosophila* embryo [86]. ERK waves are also EGFR-dependent and driven by a transcriptional switch relay where ERK activity promotes Rhomboid transcription (a protease essential for EGFR ligand activation and secretion), hence increasing ligand secretion and ERK/Rhomboid activation in the neighbouring cell. These waves promote polarised MyoII recruitment and tracheal pit invagination. Here, the range of propagation is defined by the domain of expression of the transcription factor Trachealess. Importantly, this transcriptional-driven propagation occurs at a relatively slower speed compared to the mechanochemical wave described above (1 cell—~ 5 µm—every 15 min, 0.33 µm/min). Similar transcription-driven long-range ERK waves were also described recently during zebrafish osteoblast regeneration [87], where slow ERK waves (1 cell per hour, 0.17 µm/min) travel along the full fish scale (10 µm) at very low frequency (1 wave every 48 h). These waves are driven by transcriptional upregulation of FGFR ligand by ERK signalling combined with delayed negative feedback (preventing back propagation) and promote scale regeneration.

Altogether, these works illustrate that long-range ERK waves are not a specificity of mechano-chemical waves and can also be triggered by alternative more classic trigger-wave mechanisms. However, their timescales are usually much slower, which is not necessarily compatible with the fast information processing required for collective migration. Thus, the relatively fast (~ minutes) ERK mechanosensitive module is instrumental for fast long-range coordination. Importantly, this characteristic time may explain why it was co-opted for long-range coordination of cell migration.

### Self-organisation and emergence of complex spatio-temporal pattern of cell fate

So far, we have described one example of complex spatio-temporal pattern (waves) which modulate cell migration. ERK mechanosensing is also involved in self-organised properties that will affect cell fate and tissue shape. For instance, the emergence of leader cells during MDCK collective cell migration is driven by persistent ERK activation which is mediated by the positive feedback between cell lamellipodia and HGF receptor (MET) activation, thus connecting border localisation with fate specification [54].

ERK modulation by geometry was also involved more recently in lung branching morphogenesis [49]. Using live imaging of ERK activity in ex vivo culture of mice lung, the authors found a striking correlation between epithelial curvature and ERK activity. Convex regions are associated with a higher basal surface which promotes the binding of FGF10 produced by the mesenchymal cells. Subsequently, ERK activity in epithelial cells promotes apical actin polymerisation, which extends apical area and flattens locally the tissue. As a result, two new curved regions emerge on the lateral side which through ERK activation and actin polymerisation initiate two new branches (Fig. 3B). Thus, the modulation of ERK activity by curvature is sufficient to trigger repetitive morphological patterns. Whilst the impact of ERK response delay was not formally discussed in this model, the persistence of cell curvature (regulated by the turnover of F-actin) has to be in a well-controlled range to achieve proper branching morphogenesis. These results expand the repertoire of self-organised morphogenetic processes which, through ERK response to curvature and ERK-dependent regulation of actin, can produce repetitive branching.

Whilst so far we have mostly covered the role of ERK in self-organised morphogenesis, ERK has also been recently involved in the regulation of complex spatio-temporal pattern of cell death with essential functions in tissue homeostasis. MCF10A cells can self-organise in 3D acini which form a lumen through the apoptosis of internal cells. Recently, this process was shown to be regulated by ERK pulse frequency so that external cells are protected from death through spontaneous ERK waves, whilst internal cells die because of lower frequency of ERK pulses [35]. Whilst this difference of ERK dynamics was not formally associated with ERK mechanosensing, differences in cell–cell connexion, geometry and ligand accessibility may be sufficient to trigger these frequency differences which, as such, will spontaneously regulate the spatial pattern of apoptosis and lumen formation. ERK pulses are also essential to fine-tune rate and distribution of cell death in 2D epithelia. Cell extrusion in the *Drosophila* pupal notum (a single layer epithelium) triggers transient activation of ERK in the neighbouring cells, hence inhibiting caspases and protecting transiently (for ~ 1 h) neighbouring cells from apoptosis [78]. This transient and local protection is essential to prevent the formation of clusters of cell elimination, which otherwise forms gaps and affects epithelia sealing properties (Fig. 3C). Whilst it is not yet absolutely clear whether such pulses are driven by mechanical stress, they correlate with the onset of cell extrusion (with a 5 min delay) and the stretching of the neighbouring cells and do not rely on ligand secretion from the dying cells. Importantly, this local feedback affects both the spatio-temporal distribution of cell death (where a local refractory phase follows each extrusion) as well as the total number of dying cells. A very similar process was also described in MCF10A, MDCK and NRK-52E cells, where apoptosis triggers a wave of ERK and Akt activation propagating other few cell rows [79]. This feedback tunes down the global rate of cell elimination and prevents catastrophic increase of death rate and tissue collapse upon pro-apoptotic treatment (doxorubicin or starvation). These waves require ADAM17 in the dying cell as well as EGFR, and do not rely on extracellular ligand diffusion. Whilst this was not formally tested, this could be compatible with a contribution of mechanical stress to ERK activation and propagation. Overall, these works illustrate how local ERK pulses rapidly mediated by apoptosis initiation (few minutes) are essential to redistribute cell death and prevent tissue collapse. This rapid response—faster than the characteristic time of apoptosis and extrusion induction—is essential to prevent clustered and massive cell death and may rely again on the timescale of ERK mechanosensitivity.

To conclude, ERK mechanosensitivity provides a rapid relay to adjust cell fate to the local cell environment, whether for morphogenesis and cell fate specification or tissue homeostasis. This modularity is essential for tissue plasticity and robustness in rapidly changing environment. It strongly relies on ERK modulation and relevant downstream target regulation being faster than the characteristic time of core cellular events (apoptosis, mitosis and fate specification).

### Co-optation of ERK mechanosensing during pathological conditions

We have so far described several physiological functions of ERK mechanosensitivity, from tissue homeostasis and cell death regulation, to morphogenesis and collective movements. The same modules can however also be co-opted in pathological conditions and promote tumour cell transformation and tissue invasion.

Seminal work in breast cancer cells outlined the central role of aberrant tension homeostasis in tumour progression [25, 88]. One hallmark of tumour is the formation of stiffer tissues which is regularly used for tumour diagnosis. Praszek and colleagues used a mechanical indentor to measure the mechanical properties of mammary tumours and confirmed the significant increase of stromal matrix stiffness near transformed cells and tumour [25]. Increasing matrix stiffness (by increasing collagen concentration) promotes MCF10A cell transformation, characterised by abnormal acini formation, destabilised adherens junctions and active protrusion. This transformation is driven by higher integrin clustering which through FAK activation promotes Rho and ERK phosphorylation. Moreover, EGFR concentration and phosphorylation are also increased on stiff substrate. ERK activation further enhances cell stiffness by promoting ROCK activation, thus generating a positive feedback loop promoting cell transformation. Accordingly, inhibition of EGFR, ERK, Rho or integrin is sufficient to revert the transformation phenotype on stiff matrix and restore the formation of polarised acini. Latter work confirmed this positive feedback and the central role of ERK for cell transformation, migration and proliferation [89]. Importantly, ERK depletion in MCF10A cells grown on stiff substrate was sufficient to restore 75% of the modulated transcript back to soft matrix condition levels, suggesting that ERK is a major bottleneck regulating cell transformation on stiff substrate [89]. A very similar process was recently outlined in the mouse prostate upon ablation of a subpopulation of the stromal cells at the junction between the prostate and the urethra [90]. Depletion of this Lgr5 positive population increases globally prostate stiffness, which leads to aberrant ERK activation through FAK, promotes epithelial cell turnover. However, this study did not clearly delineate whether increased stiffness was a cause or a consequence of ERK activation. These works outline the central role of focal adhesion as stiffness sensor, which through FAK can modulate ERK activity and further enhance cell contractility and cell transformation in oncogenic cells.

Other works outline alternative models of ERK modulation by mechanics during tumour progression. Squamous cell carcinoma invasiveness is also enhanced on stiffer matrix through the activation of EGFR (higher expression levels and phosphorylation) [91]. EGFR/ERK upregulates the L-type-dependent Ca^2+^ channel Cav1.1, which promotes Cdc42, Myosin light chain activation and traction forces, hence promoting collective cell migration in spheroids and mice subcutaneous grafts. Interestingly, EGFR upregulation in stiff matrix is abrogated by YAP inhibition, suggesting that EGFR mechanosensitivity is indirect in this context. Matrix stiffness also promotes glioblastoma cell proliferation through EGFR clustering in vinculin positive focal adhesion which boosts proliferation through ERK, Akt and PI3K activation [92]. Thus, ERK modulation by matrix stiffness in tumour can also be independent of FAK and rely on EGFR activation. Finally, ERK activation can also be promoted by cell autonomous increase of contractility driven by the direct modulation of actomyosin by oncogenes. Accordingly, the proliferation of pre-invasive breast cancer cells (MCF10A cells with conditional Src activation) is promoted by the upregulation of the barbed-end actin capping protein Ena/VASP like, which stabilises stress fibres and stiffen cells, hence promoting ERK activation, proliferation and further enhancing Src activation [93].

All these examples illustrate the cell-autonomous impact of ERK mechanosensitivity in tumour cells, which through a positive feedback loop between matrix stiffness, cell contractility and ERK promotes on the long-term tumour cell transformation, migration and proliferation. However, ERK mechanosensitivity can also promote tumour progression through non-autonomous effects and interactions with neighbouring healthy cells. Cell competition is a context-dependent cell elimination process which can promote the elimination of WT cells by oncogenic cells through apoptosis, cell differentiation or cell extrusion [94]. Differential sensitivity to mechanical stress can also promote the preferential elimination of one population during cell competition [95–98]. Accordingly, conditional activation of the oncogene Ras^V12^ in *Drosophila* pupal notum clones promotes cell expansion and cell death resistance, which triggers neighbouring WT cell compaction, caspase activation and cell death through EGFR/ERK downregulation [39]. This compaction-driven ERK downregulation contributes significantly to Ras clone expansion. Accordingly, activation of EGFR/ERK in WT neighbouring cells blocks their elimination and significantly slows down Ras clone expansion [39]. This work illustrates how ERK mechanosensitivity role in cell survival can be co-opted by oncogenic cells to eliminate their neighbours.

To conclude, the modulation of ERK by mechanics can also be co-opted by tumoural cells, in a cell-autonomous and non-autonomous manner, to promote their expansion and invasiveness. On the one hand, cell contractility and matrix stiffness promote ERK activation through FAK and/or EGFR which further enhance cell stiffening through Rho/ROCK activation, hence generating a positive feedback loop which promotes cell transformation, proliferation and migratory behaviours. On the other hand, tumoural cell growth can compact the neighbouring cells and trigger their elimination through compaction-driven ERK downregulation. The modulations of ERK in these pathological conditions were mostly studied over long-time scales (hours and days after mechanical stimulation). Thus, it is hard to exclude that ERK modulation may indirectly be connected to mechanics and triggered by other mechanosensitive pathways. Further work will be required to test if the complex spatio-temporal dynamics characterised in physiological contexts (see above) also play a role in tumour progression.

## Conclusion and perspectives

In this review, we illustrated multiple cellular, tissular, physiological and pathological contexts during which ERK modulation was associated with geometrical and mechanical perturbations. Whilst various perturbations and timescales were reported, vast majority were associated with relatively fast phosphorylation of ERK (few minutes) following cell stretching. The downstream activity of ERK, which can both modulate transcription and phosphorylate a large variety of substrates, offers a fast modular mechanosensitive module which can adjust rapidly several cell properties to mechanical stimuli. Thus, ERK modulation within minute time scale expands the range of mechanosensitive modules complementing systems reacting in the order of seconds (e.g.: membrane tension and mechanosensitive channels [99]) to hours (e.g. YAP/TAZ and its transcriptional outputs [100, 101]). For instance, whilst the Hippo pathway may be very relevant for long-term regulation of tissue size, growth and fate specification (taking place over hours or days) [102], ERK mechanosensitivity and its impact on the timescale of minutes help regulate fast cell fate transition, cell migration, cell division or cell death upon rapid changes of the mechanical environment, a situation very relevant to cell migration, fast morphogenesis and fluctuating environment. The combination of various mechanosensitive modules acting at different timescales is probably essential for cell and tissues to adjust their state to a complex and changing environment. In this regard, ERK modulation by mechanics plays essential roles in tissue robustness and plasticity [47, 78], can generate long-range coordination [33], and generate complex spatiotemporal pattern of shape and cell fate [49]. Cells and tissues can be exposed to mechanical stress acting on very different timescales [103]. Assessing the modularity of cell responses to these various characteristic times remains extremely challenging and is a poorly explored territory. One fundamental difficulty lies in the nested characteristic timescales of the response, which is affected, on the one hand, by the mechanosensors themselves and their sensitivity to specific speed/frequency of deformation, which on the other hand is also blurred by the intrinsic timescales/delays of the downstream pathways and their targets. Understanding how cells integrate various mechanical inputs to adapt their behaviour will probably require high multiplex imaging combining sensors of different pathways at different epistatic levels.

Whilst further work is still required to fully elucidate the molecular mechanisms connecting cell mechanical state to ERK activation, studies over the last few years point to several alternative mechanisms that may coexist and modulate ERK pathway at different epistatic levels. This is very reminiscent of the complex regulation of YAP/TAZ by mechanics, which on the one hand relies on the interaction between polarity protein, cytoskeleton and upstream regulators [104], or acts through direct modulation of its nuclear import by nucleus deformation and pore opening [105]. The Hippo pathway illustrates that various mechano-sensitive modules located at different subcellular localisations (cortex, nucleus) and responding to different key parameters (actin concentration, tension, nuclear membrane stretching) can all provide inputs to the same downstream pathway. It is as such not so surprising to find a similar complexity in the various mechanisms connecting cell mechanics to ERK modulation. Future work will help bridge these different mechanisms and study cell and tissue mechanosensing in a more integrated manner by bridging various mechanosensitive pathways/modules at different timescales in the same cells, most likely by developing a large array of live sensors and through multiplex live imaging.

## Data Availability

There are no data and no material associated with this manuscript.
